# Health monitoring among asylum seekers and refugees: a state-wide, cross-sectional, population-based study in Germany

**DOI:** 10.1186/s12982-019-0085-2

**Published:** 2019-07-07

**Authors:** Louise Biddle, Natalja Menold, Martina Bentner, Stefan Nöst, Rosa Jahn, Sandra Ziegler, Kayvan Bozorgmehr

**Affiliations:** 1 Social Determinants, Equity and Migration Group, Department of General Practice and Health Services Research, University Hospital Heidelberg, Im Neuenheimer Feld 130.3, 69120 Heidelberg, Germany; 2 Faculty of Arts, Humanities and Social Science, Institute of Sociology, Technische Universität Dresden, Dresden, Germany; 3 GESIS Leibniz Institute for the Social Sciences, Mannheim, Germany; 4 Department of Population Medicine and Health Services Research, School of Public Health, Bielefeld University, Bielefeld, Germany

**Keywords:** Health monitoring, Refugees, Asylum seekers, Survey, Healthcare access, Health status, Healthcare quality, Healthcare planning

## Abstract

**Background:**

Health monitoring in Germany falls short on generating timely, reliable and representative data among migrants, especially transient and marginalized groups such as asylum seekers and refugees (ASR). We aim to advance current health monitoring approaches and obtain reliable estimates on health status and access to essential healthcare services among ASR in Germany’s third largest federal state, Baden-Württemberg.

**Methods:**

We conducted a state-wide, cross-sectional, population-based health monitoring survey in nine languages among ASR and their children in collective accommodation centres in 44 districts. Questionnaire items capturing health status, access to care, and sociodemographic variables were taken from established surveys and translated using a team approach. Random sampling on the level of 1938 accommodation centres with 70,634 ASR was employed to draw a balanced sample of 65 centres with a net sample of 1% of the state’s ASR population. Multilingual field teams recruited eligible participants using a “door-to-door” approach. Parents completed an additional questionnaire on behalf of their children.

**Results:**

The final sample comprised 58 centres with 1843 ASR. Of the total sample expected eligible (N = 987), 41.7% (n = 412) participated in the survey. Overall, 157 households had children and received a children’s questionnaire; 61% (n = 95) of these were returned. Age, sex, and nationality of the included sample were comparable to the total population of asylum applicants in Germany. Adults reported longstanding limitations (16%), bad/very bad general health (19%), pain (25%), chronic illness (40%), depression (46%), and anxiety (45%). 52% utilised primary and 37% specialist care services in the previous 12 months, while reporting unmet needs for primary (31%) and specialist care (32%). Younger and male participants had above-average health status and below-average utilisation compared to older and female ASR.

**Conclusions:**

Our health monitoring survey yielded reliable estimates on health status and health care access among ASR, revealing relevant morbidities and patterns of care. Applying rigorous epidemiological methods in linguistically diverse, transient and marginalized populations is challenging, but feasible. Integration of this approach into state- and nation-wide health monitoring strategies is needed in order to sustain this approach as a health planning tool.

**Electronic supplementary material:**

The online version of this article (10.1186/s12982-019-0085-2) contains supplementary material, which is available to authorized users.

## Background

Due to the increased intensity, diversity, and duration of forced migration from conflict areas in the Middle East and Africa, governments of high-income countries in Europe and beyond are faced with the challenge of responding to the humanitarian needs of this population. One of these challenges is providing good access to needs-based, high-quality health care services to those whose residence status is not yet assured.

Asylum seekers and refugees (ASR) are purportedly young, male, and healthy. However, there is substantial evidence to suggest that this population has varied and nuanced health care needs due to exposure to risk factors before, during, and after flight [1, 2]. Experiences of violence, war, and torture in countries of origin, the uncertain circumstances that accompany the journey and the stressors of the asylum process and cramped living conditions in reception countries lead to a substantial burden of mental illnesses including post-traumatic stress disorder, depression, and anxiety [3–5]. A further concern is the potential for the spread of infectious disease due to crowded and precarious living conditions during and immediately after flight [6, 7]. Finally, managing chronic illnesses is becoming an increasing concern for this highly mobile population [6, 8].

In Germany, access to health care services for asylum seekers is restricted to the treatment of “acute illness and pain” during the first 15 months after arrival in Germany or until a decision has been made on the asylum claim [9, 10]. Exemptions to this rule can be made on a case-by-case basis, for example in the treatment of chronic illnesses, but the decision lies with the regional authorities, leading to uncertainties and delays in the delivery of care [11]. Asylum seekers are initially housed in reception centres, where basic medical care is typically provided in on-site clinics. Transfer to regional accommodation centres occurs between 6 weeks and several months after arrival, depending on the country of origin. Here, the provision of health services is the responsibility of regional health authorities, and asylum seekers use the same health services and structures as the resident population, albeit under the restrictions of the asylum seekers’ benefit act outlined above [10, 12].

The intensity of current forced migration globally is also accompanied by an increased diversity of ASR with regards to their reason for flight, country of origin, socio-economic status, and educational background, amongst others [13]. This diversity means that health challenges faced in any particular group of ASR will depend on its composition. Regional distribution quotas, links to diasporic networks, or differential opportunities for movement within host countries may create heterogeneous groupings under the broad umbrella of “asylum seekers and refugees”, which creates additional challenges for the provision of adequate healthcare services and structures.

This variation means that access to timely, reliable, and representative data is needed in the planning of needs-based healthcare services. In Germany and in many other European countries [14, 15], however, routine data collection is unusual in healthcare settings for ASR, and they are not included in national health monitoring structures [16]. Including ASR in health monitoring is challenging for several reasons: the population is highly mobile, the location of refugee accommodation is not centrally registered and is often safeguarded by regional authorities, the group is linguistically diverse, and may be sceptical about participating in research. Health monitoring systems are not yet prepared for these challenges, and largely fail to address them adequately [15], leading to a lack of timely and accurate data on health status and health care of ASR [16].

We report the methodology and key results of a state-wide cross-sectional, population-based survey aiming to advance health monitoring methodology and generate reliable health monitoring indicators for ASR, using Germany’s third largest federal state as an example. We address the challenges inherent in the collection of epidemiological quantitative, primary research data among this population and present methodological solutions which are feasible in the research context whilst maintaining scientific rigour.

The study aims to give a detailed report of a scientifically rigorous health monitoring survey among ASR and their children with respect to survey design, sampling and data collection. Within this aim, this study addresses the following research questions:

1. What is the health status of ASR and their children in terms of self-rated health, chronic illness, mental health, longstanding limitations, disability, quality of life, and health-seeking behaviour?
2. What is the state of care for ASR and their children in terms of access to and quality of services?

## Methods

This study was designed as a population-based, cross-sectional survey study to assess healthcare needs and access to care using established instruments. Due to the challenging research context, several methodological decisions had to be made with regard to questionnaire development, sampling, and data collection in order for the study to be feasible.

### Questionnaire development

Based on the experience gained in previous small-scale feasibility studies [17–19], a questionnaire was developed for the context of ASR using established questionnaire items and validated instruments from a variety of sources (Table 1). It was designed to be self-completed in pen and paper format. Health status was assessed using the European Health Interview Survey (EHIS; general health, pain, chronic illnesses) [20], EUROHIS quality of life scale (EUROHIS-QOL) [21], Patient Health Questionnaire-2 (PHQ-2; depression) [22], and General Anxiety Disorder-2 (GAD2) [23]. Health care utilisation was assessed using the EHIS (utilisation of primary care and specialist services), EU Statistics on Income and Living Conditions (EU-SILC; unmet needs, healthcare cost) [24], Study on the Health of Adults in Germany (DEGS; health promotion) [25], and several items from the European Health Literacy Survey (EU-HLS) [26]. Health care quality was examined by assessing the prevalence of ambulatory-care sensitive (ACSC), and as such avoidable hospitalisations [27], health system responsiveness (World Health Survey, WHS) [28], as well as by questions on medication overuse taken from the Structured Clinical Interview for DSM-5 (SCID; substance abuse) [29]. Basic socio-demographic items were taken from the DEGS and accompanied by an adapted version of the MacArthur scale on subjective social status [30], as well as several specific questions on residence status, health insurance status, and length of stay in Germany. The questionnaire comprised of a total of 104 questions prior to cognitive pretesting.

**Table 1:** Adult questionnaire items, origin, type of adaptation, translation and response characteristics

Item no.	Origin	Variable	Adapted or original	Type of adaptation	Existing or new translation	Cognitive pretest	% Missing response	Bottom/ceiling effect
1	EHIS	General health	Original	–	Translation	x	10.0%	None
2	DEGS	Health state compared to last year	Original	–	Existing (RU, GER, SER, TUR)^a^ Translation (AR, FR, FAR, ALB)		11.4%	None
3	EHIS	Chronic illness	Original	–	Translation	x	11.0%	None
4	EHIS	Limitation due to health problem	Adapted	Timeframe	Translation		15.8%	–
5	DEGS	Pain	Original	–	Existing (RU, GER, SER, TUR)^a^ Translation (AR, FR, FAR, ALB)		10.4%	None
6	EHIS	Existence of ambulatory sensitive conditions	Adapted	Revised item list	Translation		2.7%	–
7	EHIS	Avoidable hospitalisation	Adapted	Added specification	Translation		7.3%	–
8	EHIS	Other hospitalisation	Adapted	Added specification	Translation		6.8%	–
9	EHIS	Hospitalisation frequency	Adapted	Multiple choice format	Translation		12.1%	–
10	EHIS	Alcohol consumption	Original	–	Translation		7.8%	Bottom^g^
11	DEGS	Smoking	Original	–	Existing (RU, GER, SER, TUR)^a^ Translation (AR, FR, FAR, ALB)		5.6%	None
12	SCID	Medication abuse	Original	–	Translation		8.3%	–
13	EUROHIS-QOL	Quality of life	Adapted	Simplified wording	Existing (RU, GER, SER, TUR, AR, FR, FAR)^b^ Translation (ALB)	x	7.3%	None
14	EUROHIS-QOL	Quality of life	Original	–	“	x	8.5%	None
15	EUROHIS-QOL	Quality of life	Original	–	“		6.8%	None
16	EUROHIS-QOL	Quality of life	Original	–	“	x	8.0%	None
17	EUROHIS-QOL	Quality of life	Original	–	“	x	9.2%	None
18	EUROHIS-QOL	Quality of life	Original	–	“	x	11.2%	None
19	EUROHIS-QOL	Quality of life	Original	–	“		8.0%	None
20	EUROHIS-QOL	Quality of life	Original	–	“		6.3%	None
21	PHQ2	Depression	Original	–	Existing (RU, GER, SER, TUR, AR, FR)^c^ Translation (FAR, ALB)		13.1%	None
22	PHQ2	Depression	Original	–	“		13.1%	None
23	GAD2	Generalised anxiety	Original	–	Existing (RU, GER, TUR, AR, FR)^d^ Translation (SER, FAR, ALB)		11.9%	None
24	GAD2	Generalised anxiety	Original	–	“		14.0%	None
25	EHIS	Utilisation of primary, secondary and specialist healthcare services	Adapted	Added specification	Translation	x	15.1% (GP) 19.2% (specialist) 20.2% (dentist) 20.9% (psychologist)	–
26	EHIS	Frequency of utilisation of primary, secondary and specialist healthcare services	Adapted	Added specification	Translation		14.1% (GP) 20.6% (specialist) 20.2% (dentist) 23.8% (psychologist)	–
27	EHIS	Prescriptions	Original	–	Translation	x	9.5%	–
28	EHIS	Out-of-pocket payments for prescriptions	Adapted	Simplified wording	Translation	x	27.5%	–
29	EU-SILC	Unmet need primary care	Adapted	Simplified wording	Translation	x	16.8%	–
30	EU-SILC	Unmet need primary care reasons	Adapted	Added answer option	Translation	x	18.0%	–
31	EU-SILC	Unmet need specialist	Adapted	Simplified wording	Translation		20.4%	–
32	EU-SILC	Unmet need specialist reasons	Adapted	Added answer options	Translation		20.6%	–
33	DEGS	Family doctor	Original		Existing (RU, GER, SER, TUR)^a^ translation (AR, FR, FAR, ALB)		11.7%	–
34	EHIS	Utilisation of emergency care	Adapted	Added specification	Translation		8.5%	–
35	EHIS	Frequency of emergency care utilisation	Adapted	Added specification	Translation		11.9%	–
36	EPF Access to Healthcare	Financial burden of care	Original	–	Translation	x	14.3%	–
37	DEGS	Medical advice	Original	–	Existing (RU, GER, SER, TUR)^a^ Translation (AR, FR, FAR, ALB)		16.3%	–
38	DEGS	Medical advice	Original	–	Existing (RU, GER, SER, TUR)^a^ Translation (AR, FR, FAR, ALB)		15.8%	–
39	EPF Access to Healthcare	Perceived distance of medical facilities	Adapted	Added answer option	Translation		17.5% (GP) 20.9% (specialist) 12.4% (pharmacy) 17.0% (hospital)	–
40	WHS	Patient-rated quality of care	Adapted	Added answer option	Existing (GER, FR, RU, AR)^e^ Translation (TUR, SER, FAR, ALB)	x	17.5%	None
41	WHS	Patient-rated quality of care	Adapted	Added answer option	“	x	18.5%	None
42	WHS	Patient-rated quality of care	Adapted	Added answer option and specification	“	x	18.9%	None
43	WHS	Patient-rated quality of care	Adapted	Added answer option and simplified wording	“	x	21.4%	None
44	WHS	Patient-rated quality of care	Adapted	Added answer option	“	x	21.4%	None
45	WHS	Patient-rated quality of care	Adapted	Added answer option	“	x	24.3%	None
46	WHS	Patient-rated quality of care	Adapted	Added answer option	“	x	19.2%	Ceiling^g^
47	HLS-EU-16	Health literacy	Original	–	Existing (GER, AR, FAR)^f^ Translation (FR, RU, TUR, SER, ALB)	x	11.4%	None
48	HLS-EU-16	Health literacy	Original	–	“	x	14.1%	None
49	HLS-EU-16	Health literacy	Original	–	“	x	14.3%	None
50	–	Birth month	–	–	Translation		12.4%	–
51	–	Gender	–	–	Translation		10.7%	–
52	–	Nationality	–	–	Translation	x	9.7%	–
53	–	Mother tongue	–	–	Translation	x	7.8%	–
54	EHIS	School education	Adapted	Simplified language	Translation	x	14.8%	–
55	–	Professional education	–	–	Translation		15.5%	–
56	EHIS	Labour status	Adapted	Reduced answer options	Translation		19.2%	–
57	–	Number of children	–	–	Translation		10.0%	–
58	–	Family status	–	–	Translation		8.0%	–
59	–	Living with partner	–	–	Translation		14.8%	–
60	EHIS	Social networks	Original	–	Translation		11.7%	–
61	DEGS	Household size	Adapted	Added specification	Existing (RU, GER, SER, TUR)^a^ Translation (AR, FR, FAR, ALB)		13.1%	–
62	DEGS	Household income	Adapted	Reduced answer options	Existing (RU, GER, SER, TUR)^a^ Translation (AR, FR, FAR, ALB)		25.2%	None
63	–	Residence status	–	–	Translation	x	20.6%	–
64	–	Entry to Germany	–	–	Translation		16.3%	–
65	–	Number of transfers	–	–	Translation		12.6%	None
66	–	Health insurance card	–	–	Translation		12.9%	–
67	MacArthur Scale	Subjective social status	Adapted	Simplified wording, added specification	Translation	x	30.3%	None
68	MacArthur Scale	Subjective social status	Adapted	Simplified wording, added specification	Translation	x	27.4%	None

*EHIS* European Health Interview Survey, *DEGS* Study on the Health of Adults in Germany, *SCID* Structured Clinical Interview for DSM-5, *EUROHIS-QOL* European Health Interview Survey Quality of Life Scale, *PHQ2* Patient Health Questionnaire-2, *GAD2* Generalised Anxiety Disorder 2, *EU-SILC* European Survey on Income and Living Conditions, *EPF* European Patient Foundation, *WHS* World Health Survey, *HLS-EU-16* 16-item European Health Literacy Score, *AR* Arabic, *ALB* Albanian, *FAR* Farsi, *FR* French, *GER* German, *RU* Russian, *SER* Serbian, *TUR* Turkish

^a^All existing translations taken from [46], made available upon request

^b^All existing translations taken from [47], publicly available via http://depts.washington.edu/seaqol/WHOQOL-BREF

^c^GER, FR, RU, SER: [48], publicly available via https://www.phqscreeners.com/; TUR: [49] AR: [50] both made available upon request

^d^GER, RU, TUR: [51] publicly available via https://www.phqscreeners.com/; FR: [47] AR: [50], both made available upon request

^e^All existing translations taken from: [52],made available upon request

^f^GER: [53] publicly available; AR, FAR: [54] made available upon request

^g^Corresponding figures can be found in the Additional file 1: Web Appendix

A separate questionnaire about the children of ASR was developed, to be completed by their parents on their behalf. Health status and health care utilisation were assessed using questions from EHIS, DEGS, and the Study on the Health of Children and Adolescents in Germany (KiGGS) [31]. Conduct, social, and emotional development were assessed using the Strengths and Difficulties Questionnaire (SDQ) [32]. Socio-demographic questions were identical to the adult survey, but characteristics which are not applicable or identical for children and their parents (marital status, social network, family size, residence status, household size & income, subjective social status) were omitted. The children’s questionnaire comprised of 56 questions.

The questionnaire was developed in English and German and translated into seven further languages (Albanian, Arabic, Farsi, French, Russian, Serbian, Turkish) using a team approach [33]. Two independent translations were made by certified interpreters, which were subsequently synthesised in a joint meeting with both translators and the multidisciplinary research team consisting of a social scientist (LB) and an epidemiologist and trained physician (KB). Existing, validated translations of questionnaire items were checked by translators for language quality and were used wherever possible (see Table 1). Where necessary, existing translations were modified in the synthesis process to fit the overall structure of the questionnaire, ensure comparability across languages, address concerns regarding the translation quality, or suit the situation of the asylum seeker population.

A cognitive pretesting method [34] was then applied to check for understanding of several questionnaire items of the adult questionnaire with nine individuals in five languages (Arabic, English, Farsi, Russian, and Serbian). This resulted in the omission of several items and some structural changes. The content of the items remained unchanged [35]. Following these changes, a total of 68 items remained in the final version of the adult questionnaire.

### Ethical approval

Ethical approval was obtained from the ethics committee of the Medical Faculty Heidelberg on 12.10.2017 (S-516/2017).

### Sampling design

The target population of our study is defined as ASR living in collective accommodation centres in the state Baden-Württemberg. Residents were eligible for inclusion if they were 18 years old or older and spoke at least one of the nine study languages. We included individuals who could not read or write if they reported having someone who they trusted who could help them to complete the questionnaire.

Since centralised registers of refugee accommodations are not available, we listed and randomly selected accommodation centres in all districts of the German state of Baden-Württemberg, which is the third largest state in the country. ASR who reside in this type of accommodation have been quasi-randomly transferred based on an administrative quota from state reception centres into districts (corresponding to level 3 of the Nomenclature of Territorial Units for Statistics, NUTS-3). At district-level, health and social welfare of the ASR population is the responsibility of regional authorities. ASR must reside in such accommodation until their asylum claim has been processed. Entitlement to move to independent housing is granted after 15 months or after acceptance as refugee, but individuals remain in the assigned accommodation centres even longer if the search for independent housing proves to be difficult.

With permission from the state Ministry for Social Affairs and the state’s District Council, each of the regional authorities was contacted for lists of accommodation centres in each region along with number of ASR per facility. To select the sample, a balanced sampling algorithm was used with equal inclusion probabilities for all sampling units. Balanced sampling tries to balance the sample data on a set of auxiliary variables in such a way that the (weighted) sample mean and the population mean of the auxiliary variable are equal or close if equality is impossible to achieve [36].

The auxiliary variables used to balance the sample were the number of ASR residing in the collective accommodation centre and the number of ASR residing in the region. A classification of the accommodation centres was undertaken with four classes (quartiles), where the class boundaries correspond to the 0%, 25%, 50%, 75%, and 100% of the number of refugees in the accommodation centres. A classification of the geographical unit of the region according to the number of ASR was undertaken with 10 classes (deciles), where the class boundaries correspond to the 0%, 10%, 20%, […], 90%, and 100% quantiles of number of ASR in the region.

The balancing on the two categorical variables has a similar effect to stratification, but separately on both variables. The balancing on number of refugees in each accommodation centre helps to control for the number of refugees in the sample.

A random sample of 65 units from 1938 accommodation centres was drawn. The number of facilities drawn was chosen to achieve a gross sample of about 2350 ASR and approximate a net sample size of 1% of all ASR in the state, as is common practice in the German Microcensus [37], assuming a 30% response rate. Five “nearest neighbours” were chosen for each facility in the sample, representing accommodation centres that had an equal or similar probability of being included in the sample based on size and regional number of ASR (i.e. not necessarily based on geographical proximity). These were selected in cases where the sample facility had closed in the interim period between sample selection and data collection.

### Data collection

Data collection was carried out by trained, multi-lingual field teams in a door-to-door approach, meaning that the research team approached every residential room within the facility. Every accommodation centre was visited on two subsequent days, aiming to recruit all residents in each facility. Centre coordinators and/or responsible social workers were contacted at least one week ahead of time to announce purpose and time of the survey visit either in person or via multi-lingual flyers.

During data collection, all participants were approached personally by the field team, and explained the purpose of the study, voluntary participation, confidential data handling, and anonymity of results both verbally and in writing. Residents were handed a questionnaire and information sheet in one of the nine languages as well as non-monetary, unconditional incentives. In addition to multi-lingual team members, language support was given by short standardised audio messages in all study languages to explain the purpose of the research. If clarification was required, on-demand internet-based video interpreting services were available via tablets to aid communication. Participants had the choice of returning the questionnaire in person to the field team, in the post via a prepaid envelope, or by completing the questionnaire online by scanning a unique QR-code on their questionnaire with a mobile device. Field teams documented every contact with a potential participant, noting down, as required, reasons for exclusion from the study (e.g. illiteracy), reasons for refusal, language of distributed questionnaire, and sex of the individual.

Participants were also asked if they had any children under 18 years old living with them in the accommodation centre. If so, the participant was also given a proxy questionnaire for one of their children, to be completed on their behalf. We asked participants to complete the questionnaire for only one child in order to retain feasibility of the approach, reduce the questionnaire burden for participants with many children, and thus ensure data quality. We asked participants to choose the child which last had its birthday, thus choosing one child at random. Adults’ and children’s questionnaires were linked using an anonymous household identifier.

### Data Analysis

Except for empty questionnaires, all returned questionnaires were included in the analysis irrespective of completeness. The recommendations of the American Association for Public Opinion Research (AAPOR) guided the calculation of response rates [38]. For the response rate, overall records included in the analysis were compared to the total sample expected to be eligible. In order to obtain the denominator, we used data from the Federal Office for Migration and Refugees (BAMF) to adjust the total sample on the date of data collection by the proportion of individuals expected to be underage [39] and our own field data to adjust for the proportion of individuals expected to be unable to participate for reasons of language or illiteracy (for further details please refer to the Additional file 1: Web Appendix). We also calculated the participation rate (referred to as cooperation rate in AAPOR guidelines) by comparing the total records included in the analysis with the total number of contacts eligible for inclusion. Bottom- and ceiling-effects were determined for items with ordinal response scales if at least 50% of respondents had chosen the lowest or highest categories respectively.

Numbers of distributed and returned adult questionnaires were compared by sex, questionnaire language, size of accommodation centre, and urban/rural setting to check for sample and response bias. Sex, questionnaire language, and size of accommodation centre data were taken from field documentation. Districts with a population density below 150 inhabitants per km^2^ were categorised as rural, those above this value as urban, following the definition of the Federal Institute for Research on Building, Urban Affairs and Spatial Development (BBSR) [40]. Odds ratios (OR) with 95% confidence intervals (CI) were calculated to assess differences in odds of returning a questionnaire between ASR in respective categories.

The sample quality was assessed by comparing sex, age group, and nationality distributions of the sample with official statistics on asylum applications for the federal state [41]. For nationality, data on asylum applications from 2016 to 2017 (Q1–Q4) were used. For sex and age group, only statistics for one quarter of 2016 (Q1) and 2017 (Q3) were available. Statistics on asylum applications can approximate, but not entirely describe, the composition of ASR in the federal state, as these do not include those who applied for asylum pre-2016 or those who have re-applied for asylum. The comparison is further limited by the fact that certain groups (from so-called “safe” countries of origin) will not be transferred to regional accommodation until their asylum case is closed.

Descriptive statistics were used to analyse selected socio-demographic characteristics, mental and physical health status, and health care access, consisting of service utilisation and unmet medical needs, of participating adults and their children. Point-prevalences and 95% CI stratified by age, sex, rural/urban characteristics, and subjective social status were calculated for health status and health care access variables and plotted against the sample average of each outcome.

Age of participants was calculated by subtracting the month of data collection by stated month and year of birth, and categorising the result into 5-year age groups. Where participants had noted only year of birth, year mid-points were imputed. For the variables “nationality” and “mother tongue”, only those categories which included ≥ 2% of participants were specified, the remaining were treated as “other”. Education was captured by questions on highest educational attainment and highest professional education. Both variables were combined based on a points system ranging from 1 (lowest level of education) to 6 (highest level of education) (see Additional file 1: Web Appendix). Implausible and “don’t know” answers were treated as missing. Household income was equivalised using the square root of the number of household members according to the method adopted in the Luxembourg Income Study [42]. Subjective Social Status in Germany was divided into low (1–4), middle (5–6), and high (7–10) categories (30). Both PHQ2 and GAD2 scores above a cut-off value of 3 were considered to indicate depressive or anxiety disorder, respectively [22].

For the children’s questionnaire, SDQ score was categorised in “normal” (0–13), “borderline” (14–16), and “abnormal” (17–40) categories [32]. As the instrument is intended for children and adolescents aged 4–17, those outside this age group were excluded from the analysis.

Microsoft Excel 15 was used for data management and descriptive analysis of response rates, all other analyses were carried out using STATA version 15.1.

## Results

### Response rates and patterns

**Fig. 1:**
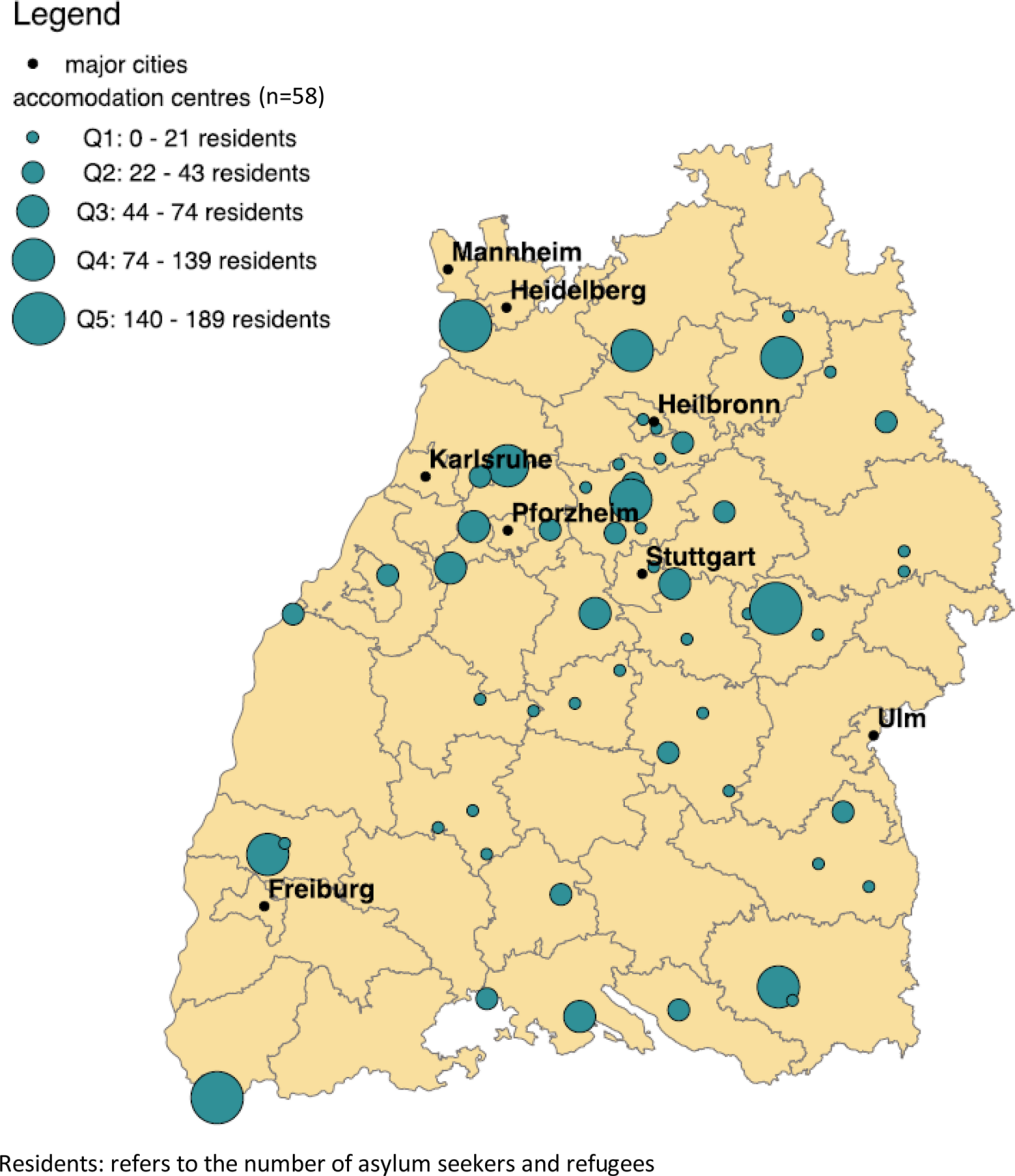
Geographic location and size of sampling units (accommodation centres), Baden-Württemberg, Germany, 2018. Residents: refers to the number of asylum seekers and refugees in each facility

A total of 65 accommodation centres were randomly selected according to the balancing algorithm (Fig. 1), including 2346 individuals at the time the sample was drawn. In 19 instances (29.2% of all facilities), a nearest neighbour facility was visited as the sample facility had closed. In seven instances (10.8% of all facilities), the chosen facility as well as all its nearest neighbours had closed, so that a total of 58 facilities were visited by the research team.

At the point of data collection, 1843 individuals resided in the visited accommodation centres (Table 2). As most facilities that had closed were small in size, the decline in the number of individuals is less a reflection of these closures, but rather attributable to an overall decline in the mean number of residents across the sample. Of the total number of individuals residing in accommodation centres, we expected 987 individuals to be eligible for inclusion in the study once age and language was taken into account, giving an eligibility rate of 56.6% (Table 2).

**Table 2:** Breakdown of sample, exclusions and survey response for adults

	N	*n*	%
Total sample drawn	2346		100
Of these: moved away or transferred/closure of accommodation centre		503	21.4
Total sample at point of data collection	1843		100
Of these: expected ineligible due to age < 18 years		745	40.4
Total adult sample at point of data collection	1098		100
Of these: expected ineligible due to language not covered by survey or illiteracy		111	10.1
*Expected sample eligible*	987		100
Of these: not reached at time of data collection		153	15.5
*Total contacts eligible for inclusion*	834		100
Of these: refusal at outset		19	2.3
Of these: questionnaire not returned		393	47.1
Total questionnaires returned	422		100
Of these: empty		10	2.4
*Total records for analysis*	412		100
*Eligibility rate* *(Expected sample eligible/Total sample at point of data collection)*			53.6
*Response rate* *(Total records for analysis/expected sample eligible)*			41.7
*Participation rate* *(Total records for analysis/total contacts eligible for inclusion)*			49.4

The team personally approached a total of 936 residents living in district accommodation centres. Eight residents (0.9%) had to be excluded on the grounds of age, 81 (8.7%) due to their language not being covered by the survey, and 13 (1.4%) because of illiteracy. Thus, 834 residents were eligible for inclusion in the study, of which 19 (2.3%) refused participation from the outset; the remainder of residents were handed a questionnaire. 157 children’s questionnaires were distributed to eligible parents without any refusals.

A total of 422 adults’ and 95 children’s questionnaires were returned to the study team, of which 63% were returned in person and 37% returned via post. This gave a total response rate of 41.7% for adults, excluding 10 questionnaires which were returned completely empty, and a participation rate of 49.4% (Table 2). For children, the participation rate was 61%. None of the participants completed their questionnaires online.

Females, residents given a questionnaire in Farsi, Arabic, or Turkish, as well as those living in facilities with fewer residents had increased odds of returning the questionnaire compared to the respective reference group. No differences in the response rate were observed between rural and urban regions (Table 3).

**Table 3:** Response rate differences by sex, survey language, accommodation size and region

	Returned^a^	Not returned^a^	Proportion returned	Odds ratio
*Sex*				
Male	253	354	0.42	Ref
Female	115	68	0.63	2.366 [1.661; 3.381]***
*Language*				
English	105	139	0.43	Ref
Albanian	10	5	0.71	2.648 [0.793; 10.139]
Arabic	113	75	0.60	1.995 [1.331; 2.992]***
German	13	23	0.36	0.748 [0.332; 1.627]
French	25	45	0.36	0.735 [0.405; 1.316]
Farsi	110	67	0.62	2.173 [1.436; 3.294]***
Russian	15	10	0.60	1.986 [0.796; 5.142]
Serbian	2	3	0.40	0.883 [0.073; 7.851]
Turkish	19	7	0.73	3.593 [1.377; 10.453]**
*Accommodation size*				
Q1 (smallest)	22	6	0.79	4.579 [1.750; 14.039]***
Q2	44	24	0.65	2.289 [1.307; 4.078]**
Q3	64	43	0.60	1.859 [1.183; 2.933]**
Q4	95	111	0.46	1.069 [0.755; 1.511]
Q5 (largest)	193	241	0.44	Ref
*Region*				
Urban	322	312	0.51	Ref
Rural	100	114	0.47	0.850 [0.615; 1.173]

Q1–Q5: quintiles, ref: Reference group

^a^Numbers may not add up to 100% due to missing data

* p < 0.05; ** p < 0.01; *** p < 0.001

**Fig. 2:**
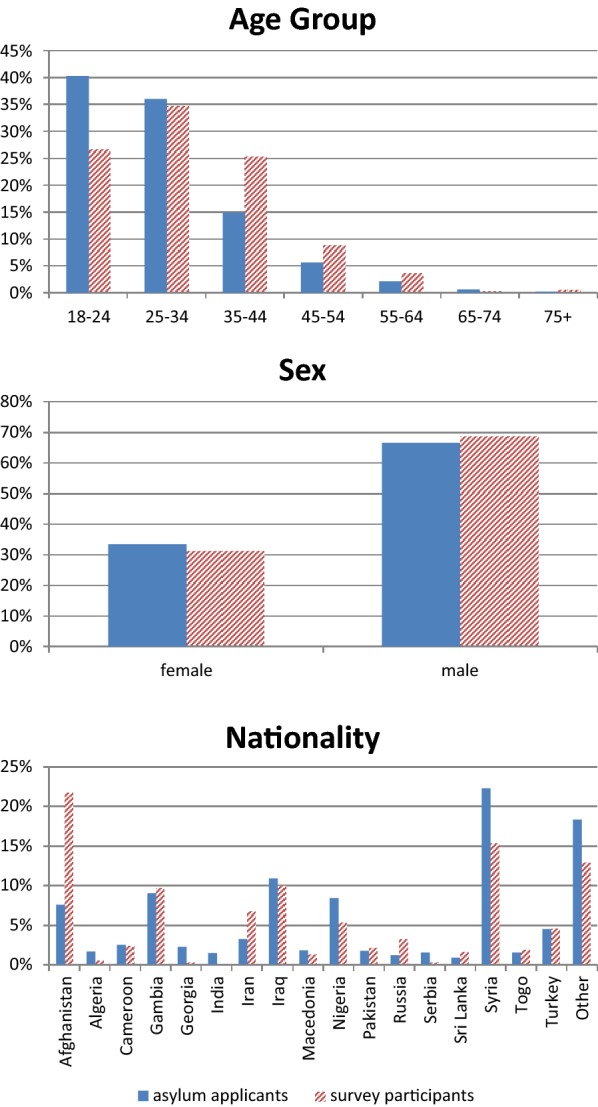
Comparison between survey participants and total asylum applicants in 2016 and 2017 by age, sex and nationality

The sample is widely comparable with the overall population of asylum applicants in the federal state related to age distribution, sex, and nationality of individuals. Only participants from Afghanistan and Syria deviate by more than five percentage-points from the distribution provided by government statistics of the state (Fig. 2).

### Study population

Participants in the study were predominantly male (68.8%) and young, with 53.2% being 30 years old or younger (Table 4). The most common nationality was Afghan (22.2%) and the most common mother tongue was Arabic (16.4%). The majority of respondents had been in Germany longer than 12 months (80.2%), with 23.6% being granted asylum in this time, 8.8% being temporarily tolerated (“Duldung”), 13.4% being rejected for asylum, and 54.5% still waiting for the outcome of their application. While 22.2% of participants reported having completed no education, 9.2% were currently in school, and 68.6% had completed at least mandatory schooling, with 24.9% of these having completed a university degree or vocational training. Although 46.4% of participants reported being unemployed, 12.9% had found work and 18.5% were currently in education. The socio-demographic characteristics of children broadly followed those of adults (Table 5), with the age of participating children approximately evenly spread across age groups.

**Table 4:** Sociodemographic characteristics of participating adult asylum seekers and refugees, Baden-Wurttemberg, Germany, 2018

	Male	Female	Total
*Age group*	*n* (%)	*n* (%)	*n* (%)
18–25	77 (32.8)	31 (27.9)	108 (31.2)
26–30	48 (20.4)	14 (12.6)	62 (17.9)
31–35	37 (15.7)	22 (19.8)	59 (17.1)
36–40	29 (12.3)	20 (18.0)	49 (14.2)
41 +	44 (18.7)	24 (21.6)	68 (19.7)
N (%)	235 (100.0)	111 (100.0)	346 (100.0)
*Nationality*	*n* (%)	*n* (%)	*n* (%)
Afghanistan	51 (20.6)	29 (25.7)	80 (22.2)
Syria	34 (13.8)	18 (15.9)	52 (14.4)
Gambia	36 (14.6)	0 (0.0)	36 (10.0)
Iraq	23 (9.3)	12 (10.6)	35 (9.7)
Iran	19 (7.7)	6 (5.3)	25 (6.9)
Nigeria	10 (4.0)	9 (8.0)	19 (5.3)
Turkey	9 (3.6)	8 (7.1)	17 (4.7)
Russia	5 (2.0)	7 (6.2)	12 (3.3)
Cameroon	7 (2.8)	2 (1.8)	9 (2.5)
Other	53 (21.4)	22 (19.5)	75 (20.8)
N (%)	247 (100.0)	113 (100.0)	360 (100.0)
*Mother tongue*	*n* (%)	*n* (%)	*n* (%)
Arabic	43 (17.6)	16 (14.0)	59 (16.4)
Dari	27 (11.0)	16 (14.0)	43 (12.0)
Kurdish	25 (10.2)	17 (14.9)	42 (11.7)
Farsi	23 (9.4)	9 (7.9)	32 (8.9)
Mandinka	24 (9.8)	0 (0.0)	24 (6.7)
Turkish	7 (2.9)	5 (4.4)	12 (3.3)
English	6 (2.4)	4 (3.5)	10 (2.8)
Tigrinya	8 (3.3)	1 (0.9)	9 (2.5)
French	4 (1.6)	2 (1.8)	6 (1.7)
Other	53 (21.6)	25 (21.9)	78 (21.7)
Multiple	25 (10.2)	19 (16.7)	44 (12.3)
N (%)	245 (100.0)	114 (100.0)	359 (100.0)
*Residence status*	*n* (%)	*n* (%)	*n* (%)
Asylum seeker	119 (55.1)	52 (53.1)	171 (54.5)
Asylum granted	49 (22.7)	25 (25.5)	74 (23.6)
Asylum status rejected	29 (13.4)	9 (9.2)	38 (12.1)
Toleration (‘Duldung’)	19 (8.8)	12 (12.2)	31 (9.9)
N (%)	216 (100.0)	98 (100.0)	314 (100.0)
*Months since arrival in Germany*	*n* (%)	*n* (%)	*n* (%)
0–6 months	5 (2.2)	4 (3.8)	9 (2.7)
6–12 months	36 (15.8)	21 (20.0)	57 (17.1)
13–15 months	62 (27.2)	26 (24.8)	88 (26.4)
16–24 months	101 (44.3)	44 (41.9)	145 (43.5)
24–36 months	24 (10.5)	10 (9.5)	34 (10.2)
N (%)	228 (100.0)	105 (100.0)	333 (100.0)
*Family status*	*n* (%)	*n* (%)	*n* (%)
Married	113 (45.6)	81 (71.7)	194 (53.7)
Single	124 (50.0)	20 (17.7)	144 (39.9)
Divorced	7 (2.8)	7 (6.2)	14 (3.9)
Partnership	4 (1.6)	3 (2.7)	7 (1.9)
Widowed	0 (0.0)	2 (1.8)	2 (0.6)
N (%)	248 (100.0)	113 (100.0)	361 (100.0)
*Subjective social status in Germany*	*n* (%)	*n* (%)	*n* (%)
Low	131 (68.6)	66 (72.5)	197 (69.9)
Medium	37 (19.4)	19 (20.9)	56 (19.9)
High	23 (12.0)	6 (6.6)	29 (10.3)
N (%)	191 (100.0)	91 (100.0)	282 (100.0)
*Subjective social status in country of origin*	*n* (%)	*n* (%)	*n* (%)
Low	77 (38.9)	38 (40.9)	115 (39.5)
Medium	49 (24.7)	32 (34.4)	81 (27.8)
High	72 (36.4)	23 (24.7)	95 (32.7)
N (%)	198 (100.0)	93 (100.0)	291 (100.0)
*Household equivalised income (quintiles)*	*n* (%)	*n* (%)	*n* (%)
€153–€265	52 (26.5)	12 (13.2)	64 (22.3)
€266–€375	52 (26.5)	13 (14.3)	65 (22.6)
€376–€459	19 (9.7)	22 (24.2)	41 (14.3)
€460–€563	31 (15.8)	28 (30.8)	59 (20.6)
€564–€3250	42 (21.4)	16 (17.6)	58 (20.2)
N (%)	196 (100.0)	91 (100.0)	287 (100.0)
*Education*	*n* (%)	*n* (%)	*n* (%)
1 (low)	41 (22.2)	33 (36.7)	74 (26.9)
2	17 (9.2)	5 (5.6)	22 (8.0)
3	33 (17.8)	17 (18.9)	50 (18.2)
4	48 (25.9)	21 (23.3)	69 (25.1)
5	34 (18.4)	8 (8.9)	42 (15.3)
6 (high)	12 (6.5)	6 (6.7)	18 (6.5)
N (%)	185 (100.0)	90 (100.0)	275 (100.0)
*Employment*	*n* (%)	*n* (%)	*n* (%)
Unemployed	107 (48.6)	41 (41.4)	148 (46.4)
Pupil or student	51 (23.2)	8 (8.1)	59 (18.5)
Working	35 (15.9)	6 (6.1)	41 (12.9)
Domestic work	1 (0.5)	39 (39.4)	40 (12.5)
Disabled	7 (3.2)	0 (0.0)	7 (2.2)
Retired	4 (1.8)	0 (0.0)	4 (1.3)
Other	15 (6.8)	5 (5.1)	20 (6.3)
N (%)	220 (100.0)	99 (100.0)	319 (100.0)

N, n: absolute frequency. Figures in brackets: column percentage

**Table 5:** Sociodemographic characteristics of children

	Male	Female	Total
*Age at interview*	*n* (%)	*n* (%)	*n* (%)
0–11 months	8 (21.1)	3 (7.0)	11 (13.6)
1–4 years	10 (26.3)	14 (32.6)	24 (29.6)
5–9 years	9 (23.7)	10 (23.3)	19 (23.5)
10–17 years	11 (28.9)	16 (37.2)	27 (33.3)
N (%)	38 (100.0)	43 (100.0)	81 (100.0)
*Months since arrival in Germany*	*n* (%)	*n* (%)	*n* (%)
0–6 months	0 (0.0)	2 (5.3)	2 (2.9)
6–12 months	13 (41.9)	7 (12.4)	20 (28.9)
13–15 months	5 (16.1)	12 (31.5)	17 (24.6)
16–23 months	10 (32.3)	14 (36.8)	24 (34.8)
24–36 months	3 (9.7)	3 (7.9)	6 (8.7)
N (%)	31 (100.0)	38 (100.0)	69 (100.0)
*Residence status (parents)*	*n* (%)	*n* (%)	*n* (%)
Asylum seeker	19 (59.4)	23 (62.2)	42 (60.9)
Asylum granted	6 (18.8)	7 (18.9)	13 (18.8)
Toleration (‘Duldung’)	3 (9.4)	2 (5.4)	5 (7.2)
Asylum status rejected	4 (12.5)	5 (13.5)	9 (13.0)
N (%)	32 (100.0)	37 (100.0)	69 (100.0)
*Subjective social status in Germany (parents)*	*n* (%)	*n* (%)	*n* (%)
Low	19 (65.5)	24 (70.6)	43 (68.2)
Middle	6 (20.7)	9 (26.5)	15 (23.8)
High	4 (13.8)	1 (2.9)	5 (8.0)
Total	29 (100.0)	34 (100.0)	63 (100.0)
*Subjective social status in country of origin (parents)*	*n* (%)	*n* (%)	*n* (%)
Bottom	13 (44.8)	17 (47.2)	30 (46.2)
Middle	10 (34.5)	8 (22.3)	18 (27.7)
Top	6 (20.7)	11 (30.5)	17 (26.1)
N (%)	29 (100.0)	36 (100.0)	65 (100.0)
*Nationality (parents)*	*n* (%)	*n* (%)	*n* (%)
Afghanistan	10 (25.6)	9 (22.0)	19 (23.8)
Cameroon	1 (2.6)	1 (2.4)	2 (2.5)
Iran	1 (2.6)	2 (4.9)	3 (3.8)
Iraq	6 (15.4)	5 (12.2)	11 (13.8)
Macedonia	1 (2.6)	0 (0.0)	1 (1.3)
Nigeria	3 (7.7)	4 (9.8)	7 (8.8)
Russia	3 (7.7)	3 (7.3)	6 (7.5)
Syria	8 (20.5)	3 (7.3)	11 (13.8)
Turkey	2 (5.1)	5 (12.2)	7 (8.8)
Other	4 (10.3)	9 (22.0)	13 (16.3)
N (%)	39 (100.0)	41 (100.0)	80 (100.0)

N, n: absolute frequency. Figures in brackets: column percentage

### Missing data and item response

Overall completeness of returned questionnaires was high, with 91% of respondents completing at least half the questionnaire, and 79% of respondents completing at least 80% of the questionnaire.

The proportion of missing data per item varied markedly between questions, from 2.7% for the presence of any chronic condition to 30.3% for the subjective social status in Germany. Highest missing items could be seen for the WHS Responsiveness and the adapted MacArthur Scales. There was, however, no trend towards increasing missing items towards the end of the questionnaire, suggesting adequate questionnaire length. Of the items with ordinal scales, the question on excessive alcohol intake exhibited a bottom effect, while the question on the cleanliness of medical facilities (WHS Responsiveness) exhibited a ceiling effect (Table 1).

### Health status

**Fig. 3:**
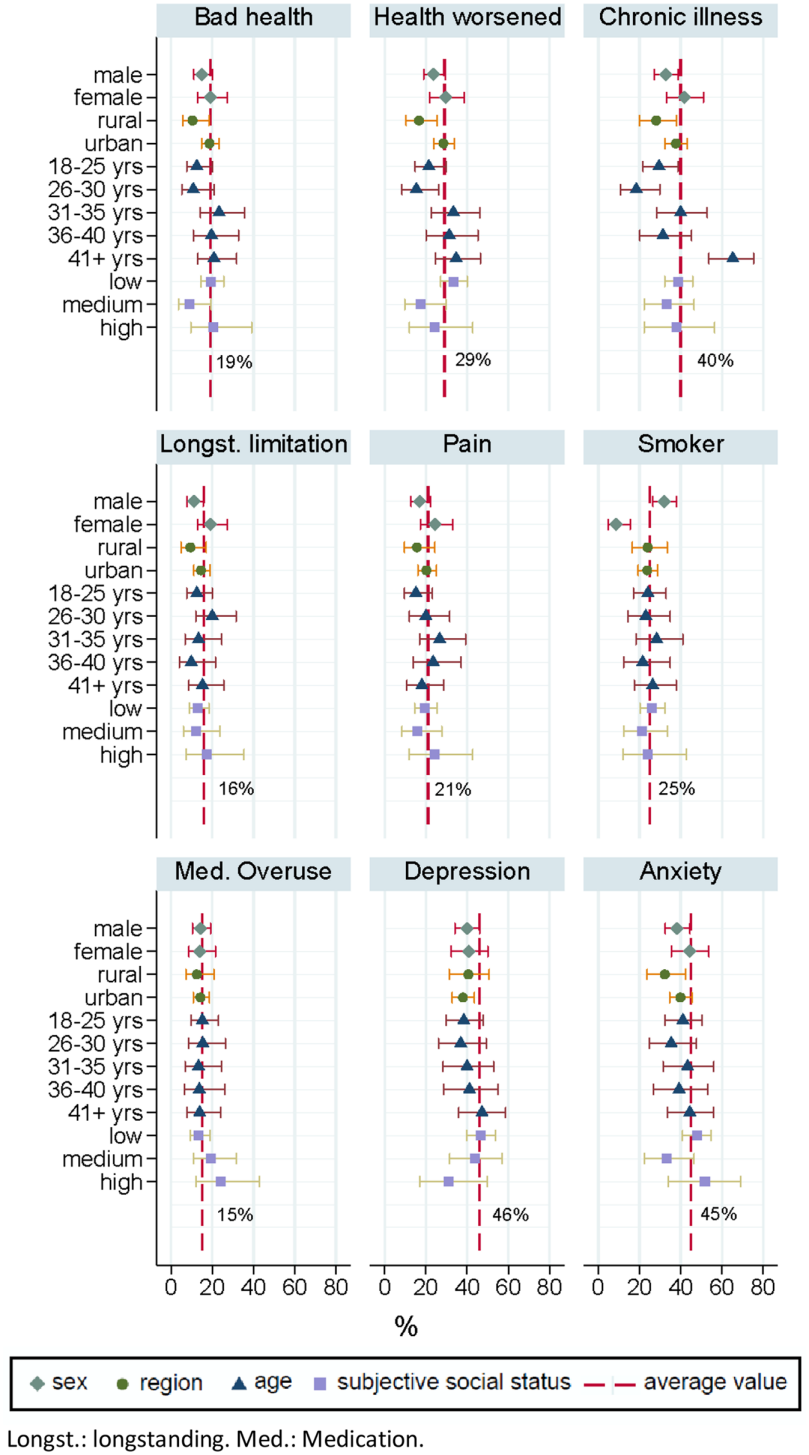
Key health status measures for adults by sex, age group, region and subjective social status. *Longst.* longstanding, *Med.* medication

An average of 19% of ASR reported “bad” or “very bad” health status, while 29% of ASR reported their health status to be worse compared to last year (Fig. 3). 40% of ASR reported living with a chronic illness, and 46% and 45% of ASR scored above the cut-off values for depression and anxiety, respectively. The prevalence of bad health, worsened health status compared to last year, chronic illnesses, longstanding limitations, pain, depression, and anxiety disorders was below average among male ASR, while the prevalence of smokers was above average. Smoking prevalence was below average in female ASR. The prevalence of bad general health status, worsened health status, longstanding limitations, and anxiety disorders was below average among ASR in rural districts. ASR in younger age groups less frequently reported bad health, worsened health, and chronic illnesses, while those in the highest age group reported above-average rates of chronic illnesses. ASR with medium subjective social status tended to report better than average self-rated health, lower health deterioration in the previous 12 months and less individuals above the cut-off for anxiety. Participants with high subjective social status appeared to show a below-average prevalence of depression.

**Fig. 4:**
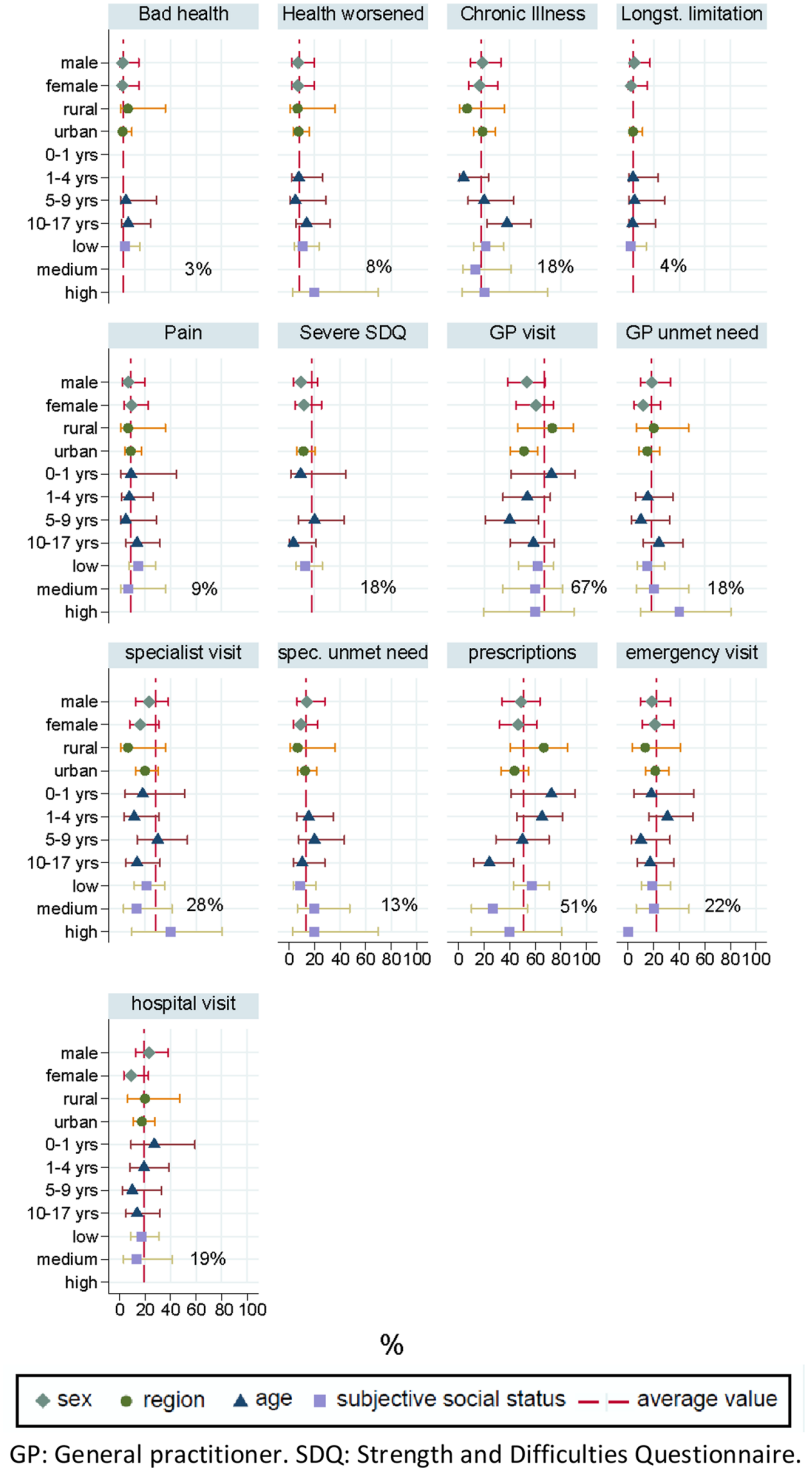
Key health status and access measures for children by sex, age group, region and subjective social status. *GP* general practitioner, *SDQ* Strength and Difficulties Questionnaire

The prevalence of bad general health was much lower in children (3%) compared to adults (19%). The same pattern was found for all other health status variables (Fig. 4). A high SDQ score was found among 18% of children, indicating high average levels of behavioural disorders. The average prevalence of any chronic illnesses among children was 18%, with above-average prevalence among the group aged 10-17 years.

### Access to health care

**Fig. 5:**
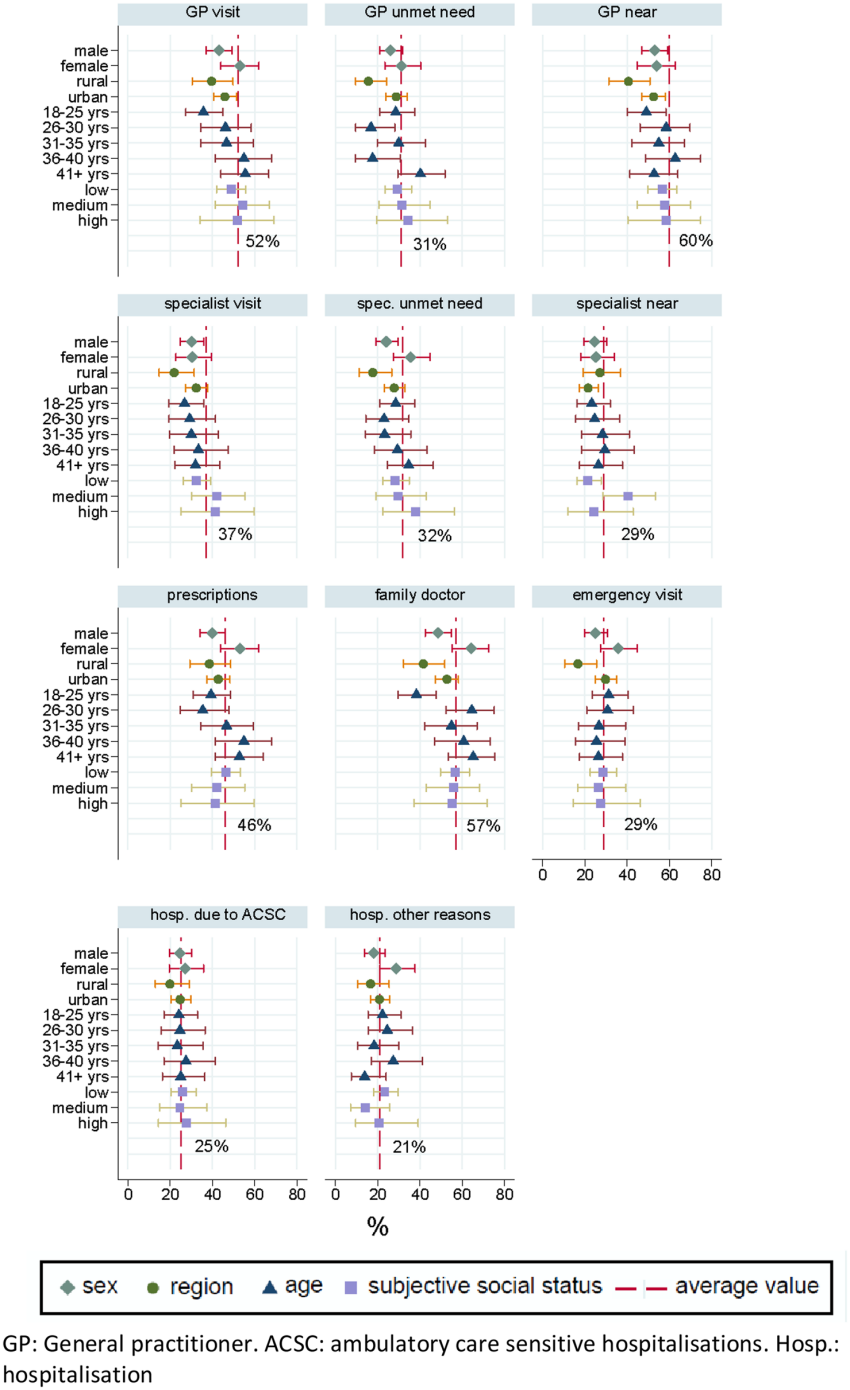
Key health access measures for adults by sex, age group, region and subjective social status. *GP* general practitioner, *ACSC* ambulatory care sensitive hospitalisations, *Hosp.* hospitalisation

Approximately half of respondents (52%) reported that they had seen a general practitioner (GP) in the last 12 months, while over a third (37%) reported they had visited a specialist in the same time period (Fig. 5). At the same time, unmet need for GP and specialist services remain high at 31% and 32% respectively. In total, 57% of ASR were registered with a family doctor. Male ASR report below average visits to the GP and the emergency room, as well as lower unmet medical need, prescriptions, and family doctor registrations. Female ASR had below average visits to a specialist, but above average unmet need of specialist services. Females also had a higher than average prevalence of prescriptions, emergency room, and hospital visits. Overall, 60% of respondents judged the proximity of their GP practice to be sufficient, while only 29% of respondents said the same about specialist services. In rural areas, respondents reported below average registration with a family doctor, utilisation of GP, specialist, emergency, and hospital services, while also less frequently judging their GP practice to be sufficiently close to their assigned accommodation centre. Younger ASR had below average visits to GPs and specialists, and average prevalence of hospitalisations, and emergency room visits (Fig. 5).

The prevalence of GP utilisation among children in the last 12 months was 67%, with below-average prevalence among asylum seeking children in urban districts and those aged 5-9 years (Fig. 4). A total of 28% of children had utilised specialist health care in the last 12 months. Unmet needs were on average higher for GP visits (18%) than for specialised care services (13%). A total of 22% of children had used emergency care services in the last 12 months, and about half of the children had received any prescription in the last 12 months (Fig. 4).

### Quality of care

An average of 25% of ASR reported having been admitted to hospital due to a condition which we categorized as ACSC, comprising heart failure, angina, heart attack, high blood pressure, bronchitis, mental health issues due to substance abuse, depression, back pain, diarrhea, influenza, ear-nose-throat infections, diabetes, epilepsy, sleep problems, or tooth cavities. This figure was slightly below average for ASR residing in rural areas, but no discernable patterns emerged for other subgroups. Use of medications in dosages higher than prescribed by a physician was reported on average by 15% of ASR and tended to be higher among those with high subjective social status (Fig. 3).

## Discussion

This study reports the methodology and results of a multi-lingual health monitoring survey among ASR in the third largest federal state in Germany. Using random sampling techniques and adaptive recruitment and surveying strategies, we were able to draw a comprehensive and dependable picture of health and health care access including quality of care among ASR and their children. We managed to obtain a reasonably high response rate and a sample which can be regarded to be of high quality, since we reached a high comparability with and representativeness for the overall population of ASR with respect to their nationality, age, and sex in the federal state under investigation.

Results showed a high burden of disease among ASR relating to self-rated health, chronic illness, and mental illness. Results related to sex and age showed consistent patterns: males tended to report above average health status and below average utilisation of services, while older subgroups demonstrated above average levels of chronic illness and above average utilisation of healthcare services. The overall unmet need for GP and specialist services among ASR is high, and the lower access to services for ASR in rural areas poses potential equity issues within the ASR population. The quality of services also warrants further investigation, as reported overuse of medications and ACSC hospitalisation prevalence is high. Further statistical analyses, including adjustment for potential confounders and use of multi-level regressions to control for the effect of clustering by accommodation centre are required to confirm these trends. Results pertaining to subjective social status appear somewhat inconsistent, with the “medium” category diverging from the other two; this also warrants further investigation. Furthermore, divergent trends for children, indicating a low burden of disease but high utilisation, need to be investigated in more detail to confirm if this is a true reflection of access to care or an artefact of the questionnaire design.

When comparing the unadjusted averages obtained for adult ASR in this study to their counterpart in the health monitoring surveys of the German general population [25], we observe that health status measures, including those with “moderate”, “bad”, or “very bad” self-rated health (25.3%), those reporting a chronic illness (36.9%), or a “medium” to “severe” longstanding limitation (34%) are considerably higher among ASR. In contrast, utilisation of health care services is higher among nationals: 96.9% of nationals report visiting any physician in the last 12 months, 79.4% visited a GP, and 71.7% visited their dentist. While stratified analyses by age, sex, and socioeconomic status are required to further investigate potential differences in health care utilisation, the crude comparisons indicate that that health care access, especially to primary care services, is lower among ASR. This is in line with previous findings from a pilot-study comparing a convenience sample of ASR with German nationals [17].

This study advances current health monitoring approaches by operationalising a sophisticated sampling strategy for a non-register based population and applying a rigorous approach translation for translation and adaptation of instruments, as well as for data collection. The study’s strengths lie in its methodological approach, which allowed us to draw a comprehensive and reliable picture of health status and health care access of ASR in district accommodation centres in the third largest German federal state. This is the first study in Germany to produce comprehensive health and health care access data using a probability sample for this heterogeneous population at a state level. The Panel of the Institute for Employment Research, Federal Office for Migration and Refugees, and the Socio-Economic Panel (IAB-BAMF-SOEP Panel, [43]), the only routine monitoring study on ASR in Germany with a probability sample, focuses on socio-economic parameters and includes only very few general health indicators and no measures of health care utilisation. Using a facility-based sampling approach, we were able to draw a random sample of all ASR in the state despite a highly mobile population and declining total numbers of ASR in the region. A further strength can be identified in the rigorous translation process with two interpreters and the research team, in which were able to discuss how to balance several translation conflicts, such as the discrepancies between formal and informal language, literal versus understandable translations, and regional language differences [44]. This study also benefitted from a cognitive pre-test [35] prior to the field study, which allowed us to address important issues with wording and translation of the survey instrument before deployment.

The response rate yielded in this study is comparable to those obtained in nation-wide surveys of the general population (e.g. 35% in the German Population Survey of the Social Sciences (ALLBUS), 42% in DEGS) [25, 45]. This was possible due to the personal contacts fostered with authorities, social workers, and ASR involved in the study. However, this approach was chosen not just due to practical, but also ethical considerations. Because individuals were personally recruited by the team in their homes, relationships of trust had to be fostered with study participants. We ensured enough time was taken to inform participants about the purpose of the study, and encouraged longer conversations to develop if these arose. We aimed for a diverse field team with regard to age, ethnicity, and gender. It must be acknowledged that the field team was not representative of the variety of backgrounds of study participants, although it could be argued that this was simply not possible given the diversity of the population. Furthermore, the spoken languages of the field team (Turkish, Farsi, Arabic) may have positively influenced response rates. Further research can investigate this potential impact.

The study team also worked very closely with regional authorities before, during, and after data collection. This was necessary to obtain the relevant information regarding the location of accommodation centres and announce our arrival to residents in advance. However, some authorities had certain prerequisites pertaining to our visit. In a few centres, for example, the field team had to be escorted by security personnel, which may have impeded the creation of a trusting relationship with residents, and in single cases may have created a pressure to participate despite the fact that field teams stressed the voluntary nature of the study. These ethical challenges need to be considered in future studies, in order to minimise negative experiences for participants during the course of the research process. Despite these limitations overall acceptance of the survey was high, and many participants appreciated the time and personal attention devoted by researchers from a university hospital to learn more about their health and health care situation.

Although we obtained a good response rate, the total sample size remains comparatively small. Our study population is broadly comparable to asylum applicants, but the small sample size entails high uncertainty in our point estimates and reduces precision, meaning that reported prevalences should be interpreted with caution. Further studies with a similar approach but with larger number of random clusters and consequently larger sample sizes are encouraged to increase precision of future estimates.

Further limitations of the study lie in the novelty of the approach: many items had to be newly translated for the study, and so the measurement equivalence of the questionnaire between languages needs to be tested. Finally, the personal approach taken in this study was labour and resource intensive: future studies should work to determine which research elements are economically viable and which may be omitted without affecting the quality of the study.

The information gathered by the methods described here can serve as a fundamental health planning tool to help regional authorities adopt a needs-based approach in health service design and delivery for the asylum seeking population. In order to sustain this approach as a health planning tool, it needs to be implemented in practice through repeat surveys at regular intervals. Ideally, these should be included in state- or nation-wide health monitoring strategies to ensure sustainability and transferability of results into practice. The approach is not restricted to the German context, and may well be adapted for use in other countries faced with the challenge of providing appropriate, needs-based services for an asylum seeking and/or refugee population.

## Conclusions

Applying rigorous epidemiological methods in linguistically diverse, transient, and marginalised populations is challenging, but feasible. We generated reliable estimates on health status and access to essential healthcare services for ASR. These indicate high levels of chronic illness and mental illness, as well as issues in access to services for rural populations and potential problems with quality of care. Given the diversity of the ASR population, a needs-based approach to health care planning must be adopted to ensure an effective and efficient delivery of services going forward. For this purpose, the methods described in this study should be implemented in state- or nation-wide health monitoring strategies. Further research should consider how health monitoring can be implemented at scale whilst retaining the necessary balance between scientific rigour and a personal, flexible approach.

## Additional file

**Additional file 1.** Web appendix containing further information on children's questionnaire, item responses and response rates.

## Data Availability

The datasets used and analysed during the current study are available from the corresponding author on reasonable request.

## Abbreviations

AAPOR: American Association for Public opinion Research
ACSC: ambulatory-care sensitive conditions
ALLBUS: German Population Survey of the Social Sciences
ASR: asylum seekers and refugees
BAMF: Federal Office for Migration and Refugees
BBSR: German Federal Institute for Research on Building, Urban Affairs and Spatial Development
DEGS: Study on the Health of Adults in Germany
EHIS: European Health Interview Survey
EPF: European Patient Foundation
EUROHIS-QOL: European Health Interview Survey Quality of Life Scale
EU-SILC: European Survey on Income and Living Conditions
GAD2: Generalised Anxiety Disorder – 2
GP: general practitioner
HLS-EU-16: 16-item European Health Literacy Score
IAB-BAMF-SOEP Panel: Panel of the Institute for Employment Research, Federal Office for Migration and Refugees, and the Socio-Economic Panel
KiGGS: Study on the Health of Children and Adolescents in Germany
NUTS-3: level 3 of the Nomenclature of Territorial Units for Statistics
PHQ2: Patient Health Questionnaire-2
SCID: Structured Clinical Interview for DSM-5
SDQ: Strengths and Difficulties Questionnaire
WHS: World Health Survey
