# Supplementary material for: Health monitoring among asylum seekers and refugees: a state-wide, cross-sectional, population-based study in Germany
Source: Emerg Themes Epidemiol. 2019 Jul 7;16:3. doi: 10.1186/s12982-019-0085-2 (PMC6613239; doi:10.1186/s12982-019-0085-2)
Supplement: Supplementary file 1 — Additional file 1. Web appendix containing further information on children's questionnaire, item responses and response rates. [file 12982_2019_85_MOESM1_ESM.docx]

Web Appendix

Additional Table 1: Children’s questionnaire items, origin, nature of adaptation, translation and response characteristics

| **Item no.** | **Origin** | **Variable** | **Adapted or original** | **Nature of adaptation** | **Existing or new translation** | **% Missing Response** | **Bottom/ Ceiling effect** |
| --- | --- | --- | --- | --- | --- | --- | --- |
| 1 | EHIS | general health | original | - | translation | 3.5% | none |
| 2 | DEGS | health state compared to last year | original | - | existing (RU, GER, SER, TUR)^A^  translation (AR, FR, FAR, ALB) | 12.63% | none |
| 3 | EHIS | chronic illness | original | - | translation | 4.2% | - |
| 4 | EHIS | limitation due to health problem | adapted | timeframe | translation | 15.8% | ceiling |
| 5 | DEGS | pain | original | - | existing (RU, GER, SER, TUR) ^A^  translation (AR, FR, FAR, ALB) | 3.2% | ceiling |
| 6 | KiGGS | child-specific infectious diseases | adapted | simplified answer scheme | existing (AR, RU, GER, SER, TUR) ^B^  translation (FR, FAR, ALB) | 67.42% | - |
| 7 | KiGGS | child-specific illnesses | adapted | simplified answer scheme | “ | 40.2% | - |
| 8 | SDQ | strengths and difficulties | original | - | existing ^C^ | 20.0% | ceiling |
| 9 | SDQ | strengths and difficulties | original | - | “ | 21.1% | none |
| 10 | SDQ | strengths and difficulties | original | - | “ | 23.2% | ceiling |
| 11 | SDQ | strengths and difficulties | original | - | “ | 20.0% | ceiling |
| 12 | SDQ | strengths and difficulties | original | - | “ | 22.1% | ceiling |
| 13 | SDQ | strengths and difficulties | original | - | “ | 23.2% | ceiling |
| 14 | SDQ | strengths and difficulties | original | - | “ | 21.1% | ceiling |
| 15 | SDQ | strengths and difficulties | original | - | “ | 25.3% | ceiling |
| 16 | SDQ | strengths and difficulties | original | - | “ | 23.2% | ceiling |
| 17 | SDQ | strengths and difficulties | original | - | “ | 28.4% | none |
| 18 | SDQ | strengths and difficulties | original | - | “ | 26.3% | ceiling |
| 19 | SDQ | strengths and difficulties | original | - | “ | 21.1% | ceiling |
| 20 | SDQ | strengths and difficulties | original | - | “ | 23.2% | ceiling |
| 21 | SDQ | strengths and difficulties | original | - | “ | 20.0% | ceiling |
| 22 | SDQ | strengths and difficulties | original | - | “ | 23.2% | none |
| 23 | SDQ | strengths and difficulties | original | - | “ | 25.3% | none |
| 24 | SDQ | strengths and difficulties | original | - | “ | 16.8% | ceiling |
| 25 | SDQ | strengths and difficulties | original | - | “ | 22.1% | ceiling |
| 26 | SDQ | strengths and difficulties | original | - | “ | 26.3% | ceiling |
| 27 | SDQ | strengths and difficulties | original | - | “ | 28.4% | ceiling |
| 28 | SDQ | strengths and difficulties | original | - | “ | 23.2% | none |
| 29 | SDQ | strengths and difficulties | original | - | “ | 26.3% | ceiling |
| 30 | SDQ | strengths and difficulties | original | - | “ | 26.3% | none |
| 31 | SDQ | strengths and difficulties | original | - | “ | 26.3% | none |
| 32 | SDQ | strengths and difficulties | original | - | “ | 23.2% | none |
| 33 | KiGGS | child-specific screening measures | adapted | simplified answer scheme | existing (AR, RU, GER, SER, TUR) ^B^  translation (FR, FAR, ALB) | 15.2% | - |
| 34 | - | vaccination booklet | - | - | translation | 4.2% | - |
| 35 | EHIS | utilisation of primary, secondary and specialist healthcare services | adapted | added specification | translation | 17.9% (GP)  35.9% (specialist)  27.4% (dentist)  36.8% (psychologist) | - |
| 36 | EHIS | frequency of utilisation of primary, secondary and specialist healthcare services | adapted | added specification | translation | 14.7% (GP)  36.8% (specialist)  29.5% (dentist)  37.9% (psychologist) | - |
| 37 | EHIS | prescriptions | original | - | translation | 6.3% | - |
| 38 | EHIS | OOP payments for prescriptions | adapted | simplified wording | translation | 21.1% | - |
| 39 | EU-SILC | unmet need primary care | adapted | simplified wording | translation | 12.6% | - |
| 40 | EU-SILC | unmet need primary care reasons | adapted | added answer option | translation | 11.6% | - |
| 41 | EU-SILC | unmet need specialist | adapted | simplified wording | translation | 12.6% | - |
| 42 | EU-SILC | unmet need specialist reasons | adapted | added answer options | translation | 11.6% | - |
| 43 | EHIS | hospitalisation | original | - | translation | 6.3% | - |
| 44 | EHIS | frequency of hospitalisation | adapted | closed answer format | translation | 11.6 | - |
| 45 | EHIS | utilisation of emergency care | adapted | added specification | translation | 10.5% | - |
| 46 | EHIS | frequency of emergency care utilisation | adapted | added specification | translation | 11.6% | - |
| 47 | EPF Access to Healthcare | financial burden of care | original | - | translation | 12.6% | - |
| 48 | DEGS | medical advice | original | - | existing (RU, GER, SER, TUR) ^A^  translation (AR, FR, FAR, ALB) | 13.7% | - |
| 49 | DEGS | medical advice | adapted | reduced answer options | “ | 21.2% | - |
| 50 | - | birth month | - | - | translation | 15.9% | - |
| 51 | - | gender | - | - | translation | 9.5% | - |
| 52 | - | entry to Germany | - | - | translation | 25.8% | - |
| 53 | - | number of transfers | - | - | translation | 7.4% | none |
| 54 | - | years of schooling | - | - | translation | 13.7% (home)  17.9% (Germany) | bottom (home)  none (Germany) |
| 55 | KiGGS | educational progress | adapted | added answer option | existing (AR, RU, GER, SER, TUR) ^B^  translation (FR, FAR, ALB) | 9.5% | none |
| 56 | KiGGS | educational progress | adapted | added answer option | “ | 10.5% | - |

^A^ All existing translations taken from (1), made available upon request

^B^ All existing translations taken from (2), made available upon request

^C^ All existing translations taken from (3), publicly available via <http://www.sdqinfo.com>

Legend: EHIS – European Health Interview Survey; DEGS - Study on the Health of Adults in Germany; KiGGS - German Health Interview and Examination Survey for Children and Adolescents; SDQ – Strengths and Difficulties Questionnaire; EU-SILC – European Survey on Income and Living Conditions; EPF – European Patient Foundation; AR – Arabic; ALB – Albanian; FAR – Farsi; FR – French; GER – German; RU – Russian; SER – Serbian; TUR - Turkish

Additional References:

1. Kurth B-M. Erste Ergebnisse aus der „Studie zur Gesundheit Erwachsener in Deutschland “(DEGS). 2013
2. Kamtsiuris, P., Lange, M., & Rosario, A. S. (2007). Der Kinder-und Jugendgesundheitssurvey (KiGGS): Stichprobendesign, Response und Nonresponse-Analyse. Bundesgesundheitsblatt Gesundheitsforschung-Gesundheitsschutz, 50(5-6), 547-556.
3. Goodman, R., Ford, T., Simmons, H., Gatward, R., & Meltzer, H. (2000). Using the Strengths and Difficulties Questionnaire (SDQ) to screen for child psychiatric disorders in a community sample. The British Journal of Psychiatry, 177(6), 534-539.

Additional Figure 1: Bottom effect on Item 10 (excessive alcohol intake)


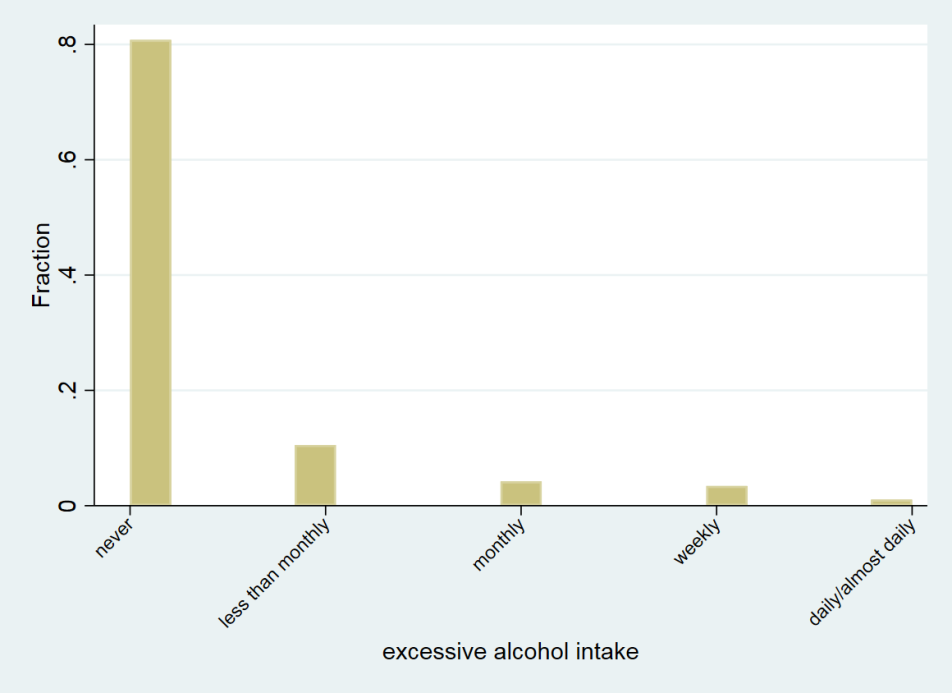


Additional Figure 2: Ceiling effect on Item 46 (patient-rated cleanliness of facilities)


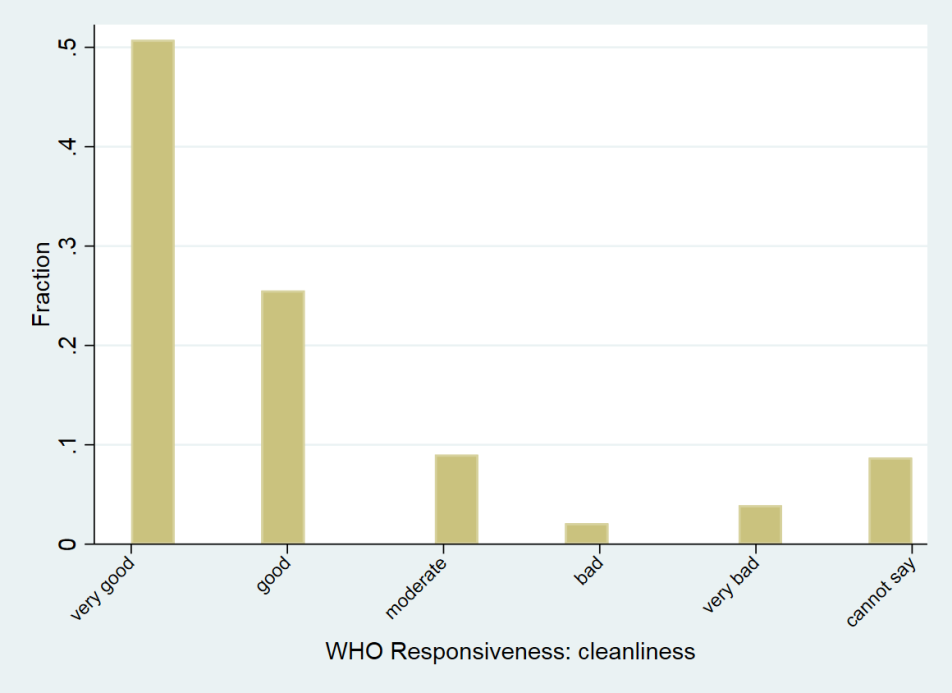


| school education | no further education | vocational training | university degree | don't know |
| --- | --- | --- | --- | --- |
| no education | 1 | 2 | missing | 1 |
| still in school | 2 | missing | missing | missing |
| mandatory schooling complete | 3 | 4 | 5 | missing |
| high school complete | 4 | 5 | 6 | missing |
| don't know | missing | missing | missing | missing |

Additional Table 2: calculation of educational score

professional education

Additional information for the calculation of the response rate

In order to calculate the response rate, the calculation of all individuals expected eligible for inclusion for our study within the sample was necessary. This is because our sample was drawn on the level of the accommodation centre, and the information we received pertaining to the numbers of individuals residing in each centre included children under 18 years of age, individuals not fluent in one of the study languages, and individuals who could not be included in the study due to illiteracy. The proportion of children in each centre was estimated by taking the number of children applying for asylum in Germany in 2017 (n=89,207) (1) and subtracting the number of unaccompanied minors for that year (n=9,084) (2), who are typically housed in separate accommodation arrangements. Thus, the number of accompanied children (n=80,123) makes up 40.4% of the total population of asylum seekers (n=198.317) (1). In order to estimate the number of individuals unable to participate due to language or illiteracy, we extrapolated data obtained from our own fieldwork (10.1% of contacts unable to participate due to language or illiteracy), under the assumption that the individuals we approached were similar to those we did not approach.

Additional references:

1. Bundesamt für Migration und Fluechtlinge.Aktuelle Zahlen zu Asyl. 2017. Available from: https://www.bamf.de/SharedDocs/Anlagen/DE/Downloads/Infothek/Statistik/Asyl/aktuelle-zahlen-zu-asyl-dezember-2017.pdf?__blob=publicationFile
2. Bundesamt fuer Migration und Fluechtlinge. Studie: Unbegleitete Minderjährige in Deutschland [Internet]. [cited 2019 Apr 09]. Available from: https://www.bamf.de/SharedDocs/Meldungen/DE/2018/EMN/20180523-am-wp80-unbegleitete-minderjaehrige.html
